# Notification that new names of prokaryotes, new combinations and new taxonomic opinions have appeared in volume 76, part 2 of the IJSEM

**DOI:** 10.1099/ijsem.0.007121

**Published:** 2026-05-29

**Authors:** Aharon Oren, Markus Göker

**Affiliations:** 1 The Institute of Life Sciences, The Hebrew University of Jerusalem, The Edmond J. Safra Campus, 9190401 Jerusalem, Israel; 2 Leibniz Institute DSMZ – German Collection of Microorganisms and Cell Cultures, Inhoffenstrasse 7B, 38124 Braunschweig, Germany

This listing of names of prokaryotes published in a previous issue of the International Journal of Systematic and Evolutionary Microbiology (IJSEM) is provided as a service to the microbiology to assist in the recognition of new names and new combinations. This procedure was proposed by the Judicial Commission [1]. The names given herein are listed according to the Rules of Priority (i.e. the time and date of publication of the articles on the journal’s platform and the order of valid publication of names in the original articles) [2].

**Table:** 

Order of article publication	Name/authors	Proposed as	Article no.	Page no.
1	*Variovorax palleresanus* Comas *et al*. 2026	sp. nov.	007057	9
2	*Clostridium botulinum* subsp. *botulinum* (van Ermengem 1896) Dif *et al*. 2026	comb. nov. [basonym: ‘*Bacillus botulinus’* van Ermengem 1896]*	007048	8
2	*Clostridium botulinum* subsp. *combesii* (Prévot and Laplanche 1947) Dif *et al*. 2026	comb. nov. [basonym: ‘*Cillobacterium combesii*’ corrig. Prévot and Laplanche 1947]*	007048	8
3	*Chitinirhabdus* Yang *et al*. 2026	gen. nov.	007035	8
3	*Chitinirhabdus sediminis* Yang *et al*. 2026	sp. nov.	007035	9
4	*Bradyrhizobium nivariense* Pulido-Suárez *et al*. 2026	sp. nov.	007059	8
5	*Allobaileyella* Deshmukh *et al*. 2026	gen. nov.	007061	1
5	*Allobaileyella intestinalis* (Wylensek *et al*. 2021) Deshmukh *et al*. 2026	comb. nov. [illegitimate basonym: *Baileyella intestinalis* Wylensek *et al*. 2021]	007061	2
5	*Allolucifera* Deshmukh *et al*. 2026	gen. nov.	007061	2
5	*Allolucifera butyrica* (Sánchez-Andrea *et al*. 2019) Deshmukh *et al*. 2026	comb. nov. [illegitimate basonym: *Lucifera butyrica* Sánchez-Andrea *et al*. 2019]	007061	2
5	*Allomicrovenator* Deshmukh *et al*. 2026	gen. nov.	007061	2
5	*Allomicrovenator marinus* (Wang *et al*. 2022) Deshmukh *et al*. 2026	comb. nov. [illegitimate basonym: *Microvenator marinus* Wang *et al*. 2022]	007061	3
5	*Allomicrovenatoraceae* Deshmukh *et al*. 2026	fam. nov.	007061	3
6	*Oceanimonas aquatica* Park *et al*. 2026	sp. nov.	007054	7
6	*Arenibacter flavimaris* Park *et al*. 2026	sp. nov.	007054	7
7	*Streptomyces labilomyceticus* (Okami *et al*. 1963) Komaki 2026	comb. nov. [basonym: *Streptomyces albosporeus* subsp. *labilomyceticus* Okami *et al*. 1963 (Approved Lists 1980)]	007056	11
7	*Streptomyces lavendulae* subsp. *colombiensis* (Pridham *et al*. 1958) Komaki 2026	comb. nov. [basonym: *Streptomyces colombiensis* Pridham *et al*. 1958 (Approved Lists 1980)]	007056	12
7	*Streptomyces rhizosphaericus* subsp. *ignotus* (Nouioui *et al*. 2025) Komaki 2026	comb. nov. [basonym: *Streptomyces asiaticus* subsp. *ignotus* Nouioui *et al*. 2025]	007056	12
7	*Streptomyces rhizosphaericus* subsp. *rhizosphaericus* (Sembiring *et al*. 2001) Komaki 2026	comb. nov. [basonym: *Streptomyces rhizosphaericus* corrig. Sembiring *et al*. 2001]	007056	12
7	*Streptomyces antimycoticus* subsp. *mordarskii* (Kumar and Goodfellow 2008) Komaki 2026	comb. nov. [basonym: *Streptomyces mordarskii* Kumar and Goodfellow 2008]	007056	13
8	*Thaumasiovibrio clandestinus* Anum *et al*. 2026	sp. nov.	007053	8
9	*Gordonia heveisoli* Suriyachadkun *et al*. 2026	sp. nov.	007055	6
9	*Gordonia gummivorans* Suriyachadkun *et al*. 2026	sp. nov.	007055	6
10	*Sediminibacterium planctonicum* Watanabe *et al*. 2026	sp. nov.	007060	9
10	*Sediminibacterium longum* Watanabe *et al*. 2026	sp. nov.	007060	10
11	*Allobrachymonas* Deshmukh and Oren 2026	gen. nov.	007072	2
11	*Allobrachymonas chironomi* (Halpern *et al*. 2009) Deshmukh and Oren 2026	comb. nov. [illegitimate basonym: *Brachymonas chironom*i Halpern *et al*. 2009]	007072	2
11	*Allobrachymonas denitrificans* (Hiraishi *et al*. 1995) Deshmukh and Oren 2026	comb. nov. [illegitimate basonym: *Brachymonas denitrificans* Hiraishi *et al*. 1995]	007072	2
11	*Allobrachymonas wangyanguii* (Gao *et al*. 2025) Deshmukh and Oren 2026	comb. nov. [illegitimate basonym: *Brachymonas wangyanguii* Gao *et al*. 2025]	007072	2
11	*Allobosea* Deshmukh and Oren 2026	gen. nov.	007072	3
11	*Allobosea beijingensis* (Gu *et al*. 2024) Deshmukh and Oren 2026	comb. nov. [illegitimate basonym: *Bosea beijingensis* Gu *et al*. 2024]	007072	3
11	*Allobosea caraganae* (Sazanova *et al*. 2019) Deshmukh and Oren 2026	comb. nov. [illegitimate basonym: *Bosea caraganae* Sazanova *et al*. 2019]	007072	3
11	*Allobosea eneae* (La Scola *et al*. 2003) Deshmukh and Oren 2026	comb. nov. [illegitimate basonym: *Bosea eneae* La Scola *et al*. 2003]	007072	3
11	*Allobosea lathyri* (De Meyer and Willems 2012) Deshmukh and Oren 2026	comb. nov. [illegitimate basonym: *Bosea lathyri* De Meyer and Willems 2012]	007072	4
11	*Allobosea lupini* (De Meyer and Willems 2012) Deshmukh and Oren 2026	comb. nov. [illegitimate basonym: *Bosea lupini* De Meyer and Willems 2012]	007072	4
11	*Allobosea massiliensis* (La Scola *et al*. 2003) Deshmukh and Oren 2026	comb. nov. [illegitimate basonym: *Bosea massiliensis* La Scola *et al*. 2003]	007072	4
11	*Allobosea minatitlanensis* (Ouattara *et al*. 2003) Deshmukh and Oren 2026	comb. nov. [illegitimate basonym: *Bosea minatitlanensis* Ouattara *et al*. 2003]	007072	4
11	*Allobosea psychrotolerans* (Albert *et al*. 2019) Deshmukh and Oren 2026	comb. nov. [illegitimate basonym: *Bosea psychrotolerans* Albert *et al*. 2019]	007072	5
11	*Allobosea robiniae* (De Meyer and Willems 2012) Deshmukh and Oren 2026	comb. nov. [illegitimate basonym: *Bosea robiniae* De Meyer and Willems 2012]	007072	5
11	*Allobosea rubneri* (Stoll *et al*. 2024) Deshmukh and Oren 2026	comb. nov. [illegitimate basonym: *Bosea rubneri* Stoll *et al*. 2024]	007072	5
11	*Allobosea spartocytisi* (Pulido-Suárez *et al*. 2023) Deshmukh and Oren 2026	comb. nov. [illegitimate basonym: *Bosea spartocytisi* Pulido-Suárez et al. 2023]	007072	5
11	*Allobosea thiooxidans* (Das *et al*. 1996) Deshmukh and Oren 2026	comb. nov. [illegitimate basonym: *Bosea thiooxidans* Das *et al*. 1996]	007072	6
11	*Allobosea vaviloviae* (Safronova *et al*. 2015) Deshmukh and Oren 2026	comb. nov. [illegitimate basonym: *Bosea vaviloviae* Safronova *et al*. 2015]	007072	6
1	*Allobosea vestrisii* (La Scola *et al*. 2003) Deshmukh and Oren 2026	comb. nov. [illegitimate basonym: *Bosea vestrisii* La Scola *et al*. 2003]	007072	6
11	*Alloboseaceae* Deshmukh and Oren 2026	fam. nov.	007072	6
12	*Corynebacterium drakensteinense* Haines *et al*. 2026†	sp. nov.	007068	8
13	*Flavobacterium oryzagri* Kim *et al*. 2026	sp. nov.	007063	5
13	*Flavobacterium oryzicola* Kim *et al*. 2026	sp. nov.	007063	7
14	*Anoxybacillus dikiliensis* Cihan *et al*. 2026	sp. nov.	006966	14
15	*Pseudomonas vesperimontis* Muratova *et al*. 2026	sp. nov.	006999	10
15	*Pseudomonas viridinivis* Muratova *et al*. 2026	sp. nov.	006999	10
16	*Reella* Christensen *et al*. 2026	gen. nov.	007067	6
16	*Reella acinonychis* Christensen *et al*. 2026	sp. nov.	007067	6
17	*Salinimicrobium aquimaris* Wan *et al*. 2026	sp. nov.	007064	8
18	*Saccharothrix sabaoui* Bellouti *et al*. 2026	sp. nov.	007030	9
19	*Pseudidiomarina xizangensis* Liu *et al*. 2026	sp. nov.	007073	8
19	*Terrihabitans aquatilis* Liu *et al*. 2026	sp. nov.	007073	8
19	*Terrihabitans deserti* (Dong *et al*. 2019) Liu *et al*. 2026	comb. nov. [basonym: *Flaviflagellibacter deserti* Dong *et al*. 2019]	007073	9
20	*Nesterenkonia maliinae* Salinas-García and Priemé 2026	sp. nov.	007062	10
20	*Arthrobacter arnarulunnguaqae* Salinas-García and Priemé 2026	sp. nov.	007062	10
21	*Serinicoccus shuyuelongi* Liu *et al*. 2026	sp. nov.	007079	9
21	*Ornithinimicrobium jinqii* Liu *et al*. 2026	sp. nov.	007079	9
22	*Pectobacterium sinaloense* Valdez-López *et al*. 2026	sp. nov.	007076	11
23	*Flavobacterium psychraerolatum* Machin *et al*. 2026	sp. nov.	007069	6
23	*Flavobacterium aeripolare* Machin *et al*. 2026	sp. nov.	007069	7
23	*Flavobacterium gelidicaeli* Machin *et al*. 2026	sp. nov.	007069	8
24	*Fulvimarina fulva* Lee *et al*. 2026	sp. nov.	007082	7
24	*Fulvimarina thalattae* Lee *et al*. 2026	sp. nov.	007082	7
25	*Pseudomonas stagnisoli* Hasan *et al*. 2026	sp. nov.	007075	8
25	*Pseudomonas cimarronensis* Hasan *et al*. 2026	sp. nov.	007075	9
26	*Halosubterraneus* Ding *et al*. 2026	gen. nov.	007081	7
26	*Halosubterraneus shenae* Ding *et al*. 2026	sp. nov.	007081	7
27	*Guggenheimellaceae* Fisher *et al*. 2026	fam. nov.	007084	8
27	*Halotolerantifilum* Fisher *et al*. 2026	gen. nov.	007084	8
27	*Halotolerantifilum yawarlongkerapense* Fisher *et al*. 2026	sp. nov.	007084	9
28	*Deinococcus pantiae* Jiya *et al*. 2026	sp. nov.	007071	5
29	*Methylobacterium synurae* Yu *et al*. 2026	sp. nov.	007077	8
30	*Methanocaldococcus abyssi* L'Haridon and Nesbø 2026	sp. nov.	007088	5
31	*Hominimerdicola intestinalis* Hisatomi *et al*. 2026	sp. nov.	007091	7
31	*Ruminococcus sporogenes* Hisatomi *et al*. 2026	sp. nov.	007091	8
31	*Hominimerdicola alba* (Hungate 1957) Hisatomi *et al*. 2026	comb. nov. [basonym: *Ruminococcus albus* Hungate 1957 (Approved Lists 1980)]	007091	8
32	*Carbonicoccus* Yan *et al*. 2026	gen. nov.	007070	12
32	*Carbonicoccus zhangzhihongae* Yan *et al*. 2026	sp. nov.	007070	12
32	*Carbonicoccus niuqiaonis* Yan *et al*. 2026	sp. nov.	007070	12
33	*Bdellovibrio bacteriopascens* González-Mireles *et al*. 2026	sp. nov.	007078	9
34	*Cohnella baijiui* Li *et al*. 2026‡	sp. nov.	007066	7
35	*Occallatibacter bavaricus* Huber *et al*. 2026	sp. nov.	007086	8
35	*Occallatibacter gabretensis* (García-Fraile *et al*. 2016) Huber *et al*. 2026	comb. nov. [basonym: *Terracidiphilus gabretensis* García-Fraile *et al*. 2016]	007086	10
36	*Hubeiibacter* Xiao *et al*. 2026	gen. nov.	007092	8
36	*Hubeiibacter jixunmingi* Xiao *et al*. 2026	sp. nov.	007092	10
37	*Bifidobacterium catenulatum* subsp. *puerorum* Orihara *et al*. 2026	subsp. nov.	007074	11

*The homotypic synonym *Clostridium botulinum* (van Ermengem 1896) Bergey *et al*. 1923 (Approved Lists 1980) was assigned to Risk Group 2 [German TRBA 466, ‘Einstufung von Prokaryonten (Bacteria und Archaea) in Risikogruppen’].

†The protologue states that the type strain was deposited as NCTC 15058, which is the type strain of *Mycobacterium antarcticum*. The correct NCTC number is 15084. Therefore, the name *Corynebacterium drakensteinense* cannot be considered validly published.

‡The etymology is given at the end of the protologue, and culture collection numbers are found in the abstract.

The following taxonomic opinions (i.e. the emendation of circumscriptions or the creation of synonyms) do not affect the status of a name under the International Code of Nomenclature of Prokaryotes. These taxonomic opinions can neither be considered as approved nor disapproved by the International Committee on Systematics of Prokaryotes.

**Table:** 

Name/authors:	Article no.	Page no.
*Clostridium baratii* corrig. (Prévot 1938) Holdeman and Moore 1970 (Approved Lists 1980)* emend. Dif *et al*. 2026	007048	8
*Clostridium colicanis* Greetham *et al*. 2003 pro synon. *Clostridium thermopalmarium* Soh *et al*. 1991	007048	8
*Clostridium estertheticum* subsp. *laramiense* (Kalchayanand *et al*. 1993) Spring *et al*. 2003 pro synon. *Clostridium estertheticum* Collins *et al*. 1993 emend. Spring *et al*. 2003	007048	8
*Clostridium nitritogenes* (Prévot 1940) Bernard *et al*. 2018* pro synon. *Clostridium baratii* corrig. (Prévot 1938) Holdeman and Moore 1970 (Approved Lists 1980)*	007048	8
*Clostridium thermopalmarium* Soh *et al*. 1991 emend. Dif *et al*. 2026	007048	8
*Clostridium estertheticum* Collins *et al*. 1993 emend. Spring *et al*. 2003 emend. Dif *et al*. 2026	007048	9
*Occallatibacter* Foesel *et al*. 2016 emend. Huber *et al*. 2026	007086	10
*Streptomyces albaduncus* Tsukiura *et al*. 1964 (Approved Lists 1980) pro synon. *Streptomyces griseoloalbus* (Kudrina 1957) Pridham *et al*. 1958 (Approved Lists 1980)	007056	11
*Streptomyces antimycoticus* subsp. *sporoclivatus* (Preobrazhenskaya 1986 ex Krassilnikov 1970) Nouioui *et al*. 2025 pro synon. *Streptomyces antimycoticus* subsp. *antimycoticus* (Waksman 1957) Nouioui *et al*. 2025	007056	11
*Streptomyces asiaticus* Sembiring *et al*. 2001 pro synon. *Streptomyces rhizosphaericus* corrig. Sembiring *et al*. 2001	007056	11
*Streptomyces colombiensis* Pridham *et al*. 1958 (Approved Lists 1980) emend. Komaki 2026	007056	12
*Streptomyces gancidicus* Suzuki 1957 (Approved Lists 1980) pro synon. *Streptomyces pseudogriseolus* Okami and Umezawa 1955 (Approved Lists 1980)	007056	11
*Streptomyces labedae* Lacey 1987 pro synon. *Streptomyces griseoincarnatus* (Preobrazhenskaya *et al*. 1957) Pridham *et al*. 1958 (Approved Lists 1980)	007056	11
*Streptomyces lavendulae* subsp. *grasserius* (Kuchaeva *et al*. 1961) Pridham 1970 (Approved Lists 1980) pro synon. *Streptomyces colombiensis* Pridham *et al*. 1958 (Approved Lists 1980)	007056	11
*Streptomyces rhizosphaericus* corrig. Sembiring *et al*. 2001 emend. Komaki 2026	007056	12
*Thaumasiovibrio* Amin *et al*. 2018 emend. Anum *et al*. 2026	007053	8

*The organism was assigned to Risk Group 2 [German TRBA 466, ‘Einstufung von Prokaryonten (Bacteria und Archaea) in Risikogruppen’].

## Abbreviations

IJSEM: International Journal of Systematic and Evolutionary Microbiology
